# Medical and Physical Intelligence

**Published:** 1803-12-01

**Authors:** 


### MEDICAL AND PHYSICAL INTELLIGENCE.

*A Letter from Mess. Paul and Harfield to the Editors, dated Villiers Street, Strand, Nov.* 10, 1803, *contains the following Information.*

### On Artifical Cheltenham Water.

Since the publication of the "Reports on the Preparation of Artificial Mineral Waters," which were circulated at the opening of our manufactory in the spring of 1802, we have had the satisfaction to see our new establishment patronized, in the first instance, by the most eminent men in the several departments of medical science, and soon afterwards greatly encouraged by an enlightened public. During this space of time, several hints have been given to us, and various improvements have been suggested in the preparation of mineral waters by medical men of science and respectability, which have enabled us in some instances to extend still farther this class of remedies.

As it is our intention, at some future period, to give the public a full account of these improvements, we shall not at present enter into any particulars respecting them. But as there is one, which has already undergone a sufficient trial to enable us to speak with some confidence of its utility, we beg leave to submit it immediately to the consideration of the faculty.

Some sort of artificial mineral water, uniting under a pleasant form the tonic qualities of a chalybeate with those of a laxative medicine (such as is met with in the Cheltenham water) has long been sought for as a valuable acquisition. Natural waters of this kind not admitting of being transported without losing some of their properties, owing chiefly to a loss of gas, and a precipitation of iron, various substitutes have been proposed; the most effectual of which consists in preparing, in the form of a powder, the solid ingredients of the spring, and dissolving them in water at the moment of their being used. But it is obvious that in this imitation, useful as it may be, the medicine is deprived of its gaseous acid, which serves to dissolve and suspend the iron, and contributes greatly to render this combination effectual as well as palatable.

These advantages we flatter ourselves to have fully obtained, by combining together those two kinds of water, which are mentioned in our reports under the names of *Seidlitz* and *Spa*, so as to impregnate by means of our particular processes each pint of water with the following ingredients:

Carbonic acid, 5 times its volume.
Carbonate of iron, 1 grain.
Magnesia vitriolata, 6 drachms.

This water, we are authorised to say, has been found upon trial to be remarkably pleasant, and has appeared beneficial in the hands of some eminent physicians in cases in which Cheltenham waters would have been deemed adviseable. This mode of impregnation, pregnation, we need hardly observe, has the additional advantage of enabling us to vary the proportion either of the iron or neutral salt, as circumstances may require, and of covering almost entirely the bitter taste of the latter, by increasing greatly the proportion of the gaseous acid. Yet in recommending to notice this new preparation, we are far from imagining that those patients who can resort to the natural spring may not derive material advantages from a number of favourable circumstances connected with such excursions: but we submit to the consideration of the faculty how far the water itself, from the circumstances just mentioned, may be so prepared as to be at least as effectual, and often even better adapted to the constitution, and particular state of patients, as that which is drank on the spot itself. As the Seidlitz water when mixed with Spa in the manner used at our establishment, may be transported to almost any distance without injury, we would, with great diffidence, suggest the probable advantage of sending it to such climates as give rise to those diseases which require the combined powers of saline purgatives with tonics for their cure."

---

The history of an appearance, called in French the Blue Disease, (Maladie Blue) or that blueish livid appearance arising from malconformation of the heart, or the great vessels carrying on the circulation, was read at the Medical Society of Nancy, of which the author, M. Thiebaut, is a member. The child, a boy, was two years of age, and was remarkable for the deep colour of the skin throughout the body; the child was frequently attacked with epilepsy, the returns of which increased and terminated his existence. The author had no opportunity of examining the body, we therefore presume that the cause would be found the same as that described by Morgagni, Hunter, &c. The author conceives, that the disease may arise in the first instance, from a plethoric state of the infant at birth, preventing the new changes that should take place in the circulation, and suggests the propriety of letting a certain quantity of blood from the umbilical cord immediatcly after birth.

---

Mr. Renaud thinks that arsenic in a metallic state has no effect on the animal body; an opinion to which he was led by analogy, and by some experiments. Metals in general seem only to act on the animal body, when in a state of oxydation; hence all metals which are not easily oxydable, have but little or no effect on the living body. Thus quicksilver, as a metal, has not the least action but only a mechanical one, depending on its natural heaviness; whereas, in an oxydated state, it proves strongly stimulating, and often corrosive. It is however attended with many difficulties to make experiments with metallic arsenic, this metal being already oxydable by the mere contact of the air, besides which the slightest trituration is capable of oxydating it, on which account it cannot be given in powder, and in pieces it will only act with its surface, because it dissolves with great difficulty in the stomach. For these reasons Mr. R. took the arsenial mundic, which may be reduced reduced to a fine powder without losing its metallic gloss. Mr. R. gave of this substance from eighteen grains to two drachms to dogs, without perceiving any symptoms of a deleterious effect.

---

Dr. Deiman, of Amsterdam, employed the suroxygenated muriatic acid mixed with oil as one of the best external remedies in scabies and other cutaneous diseases, and he succeeded in curing a very obstinate species of scabies with a liniment, in which sixty drops of the acid were added to one ounce of oil. He used this remedy with equal success in tinea capitis, and herpetic complaints of the most obstinate kind. Dr. Binkman, of the same place, praises likewise this combination, but in tinea capitis he advises to add more acid. The oil is mixed with the acid by shaking it in a glass vessel, which is well closed. No more than one ounce ought to be prepared at once. The vessel, in which the liniment is preserved, must be put in a dark place, and be shaken, before it be applied.

---

Dr. Ackermann, of Mentz, has made several Galvanic experiments on the body of a beheaded person a quarter of an hour after decapitation. The battery which he used consisted of 100 strata of zinc and copper plates. A small sponge, moistened with a solution of ammonia, and connected with the hydrogen pole, being introduced into the intestinum rectum, another sponge was laid on the wound of the neck, and the head placed above it, into the right ear of which a small sponge had been put, tied to a wire, whose end was held in the hand. On touching the oxygen pole of the battery, all the muscles of the body were thrown into violent convulsions, the spine lifting itself and bending repeatedly; both arms, forcing themselves out of the hands of the assistant, were forcibly drawn towards the body; the muscles of the face were likewise convulsed, and the masticatories opened and closed the jaws with gnashing of the teeth. In the experiments which were made on the body alone, the muscles of the extremities were much convulsed; and in those made on the head only, the eye-balls were violently rolled within their cavities. All these contractions continued above an hour; and even when the body had received the temperature of the atmosphere, slight contractions could be perceived.

---

Edinburgh Classes. Mr. Charles Bell is now delivering his Course of Lectures under the following divisions, Anatomy, Pathology, and Surgery. The Course will conclude early in April.

---

Dr. Batty will begin his Second Course of Lectures on the Theory and Practice of Midwifery, &c. on Monday, Jan. 2, 1804.

